# Defining Global Neuroendocrine Gene Expression Patterns Associated with Reproductive Seasonality in Fish

**DOI:** 10.1371/journal.pone.0005816

**Published:** 2009-06-05

**Authors:** Dapeng Zhang, Huiling Xiong, Jan A. Mennigen, Jason T. Popesku, Vicki L. Marlatt, Christopher J. Martyniuk, Kate Crump, Andrew R. Cossins, Xuhua Xia, Vance L. Trudeau

**Affiliations:** 1 Centre for Advanced Research in Environmental Genomics (CAREG), Department of Biology, University of Ottawa, Ottawa, Ontario, Canada; 2 School of Biological Sciences, University of Liverpool, Liverpool, United Kingdom; Pennsylvania State University, United States of America

## Abstract

**Background:**

Many vertebrates, including the goldfish, exhibit seasonal reproductive rhythms, which are a result of interactions between external environmental stimuli and internal endocrine systems in the hypothalamo-pituitary-gonadal axis. While it is long believed that differential expression of neuroendocrine genes contributes to establishing seasonal reproductive rhythms, no systems-level investigation has yet been conducted.

**Methodology/Principal Findings:**

In the present study, by analyzing multiple female goldfish brain microarray datasets, we have characterized global gene expression patterns for a seasonal cycle. A core set of genes (873 genes) in the hypothalamus were identified to be differentially expressed between May, August and December, which correspond to physiologically distinct stages that are sexually mature (prespawning), sexual regression, and early gonadal redevelopment, respectively. Expression changes of these genes are also shared by another brain region, the telencephalon, as revealed by multivariate analysis. More importantly, by examining one dataset obtained from fish in October who were kept under long-daylength photoperiod (16 h) typical of the springtime breeding season (May), we observed that the expression of identified genes appears regulated by photoperiod, a major factor controlling vertebrate reproductive cyclicity. Gene ontology analysis revealed that hormone genes and genes functionally involved in G-protein coupled receptor signaling pathway and transmission of nerve impulses are significantly enriched in an expression pattern, whose transition is located between prespawning and sexually regressed stages. The existence of seasonal expression patterns was verified for several genes including isotocin, ependymin II, GABA_A_ gamma2 receptor, calmodulin, and aromatase b by independent samplings of goldfish brains from six seasonal time points and real-time PCR assays.

**Conclusions/Significance:**

Using both theoretical and experimental strategies, we report for the first time global gene expression patterns throughout a breeding season which may account for dynamic neuroendocrine regulation of seasonal reproductive development.

## Introduction

Fundamental to the survival of most organisms, be they yeast, plants, fishes, or mammals, are biological rhythms with periodic (daily, monthly or annual) changes in behaviour and physiology [1]. The daily circadian rhythm is exemplified by opening/closing of flowers or the daily sleep cycle in humans. The menstrual cycle of women is a typical monthly rhythm whereas circannual rhythms include bird migrations, hibernation in frogs and mammals. Marked reproductive seasonality in numerous vertebrate classes, including fish, ensures that reproduction and subsequent development of offspring is coordinated with optimal environmental and nutritional conditions. It has long been accepted that external environmental influences such as photoperiod [2], [3], [4] and temperature [4], [5], [6] exert dominant roles in biological rhythms, and internal neuroendocrine systems such as the pineal gland, hypothalamus and pituitary coordinate these signals [7], [8], [9], [10]. In this study, we use theoretical and experimental approaches to better understand global genomic regulation of the neuroendocrine system during seasonal reproduction.

The bony fishes or teleosts represent more than half of all vertebrates. Numerous characteristics of goldfish (*Carassius auratus*), a member of the family Cyprinidae, one of the largest vertebrate families, make this species an excellent model for neuroendocrine signaling and the control of seasonal reproduction [11], [12]. Similar to many teleosts, goldfish employ a seasonal reproductive strategy [5] characterized by distinct stages. In temperate regions of the Northern hemisphere the exact timing of these stages in goldfish depends on geographical location but can be generally classified as follows: gonadal regression (June-October), recrudescence or redevelopment (October-March), prespawning (April-May) and spawning or breeding season (May or early June).

Seasonal reproductive cyclicity including annual regeneration of gonadal tissues is mediated by a hormone regulatory pathway primarily involving gonadotropin-releasing hormone (GnRH), luteinizing hormone (LH), FSH, growth hormone (GH), melatonin, and sex steroid hormones [8], [9], [10]. The main hypophysiotrophic systems controlling LH and GH are located in the hypothalamus and the preoptic region of the telencephalon, which have been well-described and characterized in goldfish [8], [13]. Transduction of photoperiodic signals via retino-hypothalamo-pineal pathways results in melatonin secretion patterns typically reflecting the length of the dark period [14], [15], [16]. Melatonin influences the neuroendocrine system via its receptors which are widely distributed in the teleost brain [17], [18], [19]. Multiple GnRH forms [20] activate anterior pituitary receptors to stimulate the expression and release of LH, follicle-stimulating hormone (FSH) and GH, which are all involved in controlling seasonal gonadal development and sex steroid synthesis. Moreover, synchronization of fish reproductive behaviours in the breeding season involves a well-defined sex pheromone response system, culminating in surge release of LH that induces ovulation in females and sperm release in males [21]. Testosterone (T) and estrogen (E2) exert a primary role in gonadal development locally, and have profound positive and negative feedback actions at the levels of the brain and pituitary [8]. In addition to these core members of hypothalamo-pituitary-gonadal (HPG) axis, there is complex and interactive regulation of GnRH and LH/FSH by a multitude of neuroendocrine factors including classical neurotransmitters and other neuropeptides [8], [9], [10], [12], [22]. Glutamate, gamma-aminobutyric acid (GABA), and a multitude of neuropeptides are predominantly stimulatory on LH release whereas dopamine is the single most potent inhibitor of LH release in teleosts [8], [12], [22].

It has been extensively described that reproductive seasonality in teleosts is characterized by their seasonal profiles or changes in serum hormone levels [23], [24], [25], brain hormone content [26], brain enzyme activities [23], morphological [27] and histological [28] phenotypes. More recently, there is recognition that seasonal expression of the neuroendocrine genes including neurohypophysial hormones, synthesis enzymes, and related signaling pathway proteins may be utilized to link the external environmental influences and the establishment of fish reproductive cyclicity. It has been observed that in teleosts many neuroendocrine genes including aromatase b [29], GnRH receptors [30], glutamic acid decarboxylase (GAD) [31], cholecystokinin (CCK) [32], preprotachykinin [33] and secretogranin-II [34] have seasonal expression profiles, correlating with seasonal changes in gonadal size and sex steroid serum levels [23], [24], [35], [36]. However, no study at the transcriptomic level has been conducted to investigate the global gene expression changes in fish neuroendocrine systems throughout an annual reproductive cycle. We previously utilized microarrays to identify hormone or endocrine disrupting chemical-regulated genes in the goldfish neuroendocrine system [37], [38], [39], [40]. All experiments were based on goldfish sampled at different seasonal time points; thus, these microarray datasets contain important seasonal gene expression information (Table S1). In the present study, on the basis of multiple goldfish microarray datasets, we used comprehensive normalizations, differential gene expression identification, multivariate and gene ontology (GO) analyses to characterize the season-related expression patterns in the female goldfish brain. A core gene set was identified whose expression exhibits a seasonal change with fish reproductive cycle. We illustrate that the gene expression change is regulated by photoperiod. Gene ontology (GO) analysis further revealed that neuroendocrine-related genes are significantly enriched in an expression pattern, which follows a high-low-low profile along the spring-summer-winter seasonal axis. This pattern was confirmed by a series of independent seasonal brain sampling and real-time PCR verification on five important neuroendocrine genes. We hypothesize that the seasonal expression patterns may account for dynamic neuroendocrine regulation of reproductive cyclicity.

## Results

### Extracting neuroendocrine brain gene expression information from multiple female goldfish microarray datasets

**Figure 1:**
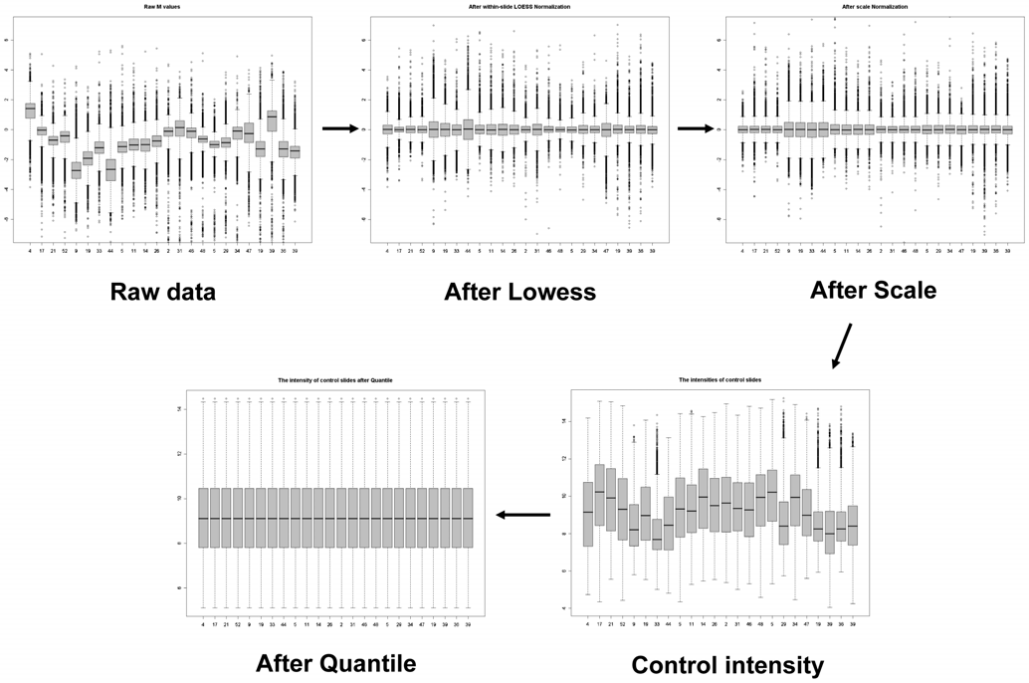
Boxplots of M values showing data distribution during the normalization procedures.

Experimental microarray data from the AURATUS goldfish environmental genomics project were used to define seasonal gene expression information (Table S1). In order to remove systemic bias and random variation within and between different experiments, we conducted a series of data transformation and normalization procedures. For each experiment, we first subjected the slides to Lowess and Scale normalizations. The raw control sample intensity was extracted and used to represent transcriptomic status of brain samples in different seasons. We further removed the bias between different experiments using Quantile normalization. This normalization procedure was firstly used for the slides (n = 24) with an mRNA source from female hypothalamus (Hyp), and Figure 1 shows data distribution for all slides during normalizations. Datasets can be divided into three seasonal time points (May, August, and December), which correspond to the distinct physiological periods of sexually mature prespawning, sexually regressed, and early redevelopment of goldfish ovary, respectively.

The global transcriptomic relationships among slides were evaluated by principal component analysis (PCA). PCA is a dimension-reducing multivariate statistical technique for simplifying complex datasets [41], [42], [43]. The results of the PCA are visualized in a two-dimensional plot. This sub-space captures the highest amount of total variability. The points in the plots represent the global transcriptomes of the different slides. The transcriptomes that are clustered together are considered overall to be similar, while more dissimilar transcriptomes are further apart. As shown in Figure S1, although from different experiments, global transcriptomes of the Hyp slides from a given season are closely clustered together. This indicates that our normalization procedure was effective in identifying the season-dependent gene expression information in these microarray datasets.

### Differentially expressed genes in female hypothalamus along the reproductive seasonal cycle

**Figure 2:**
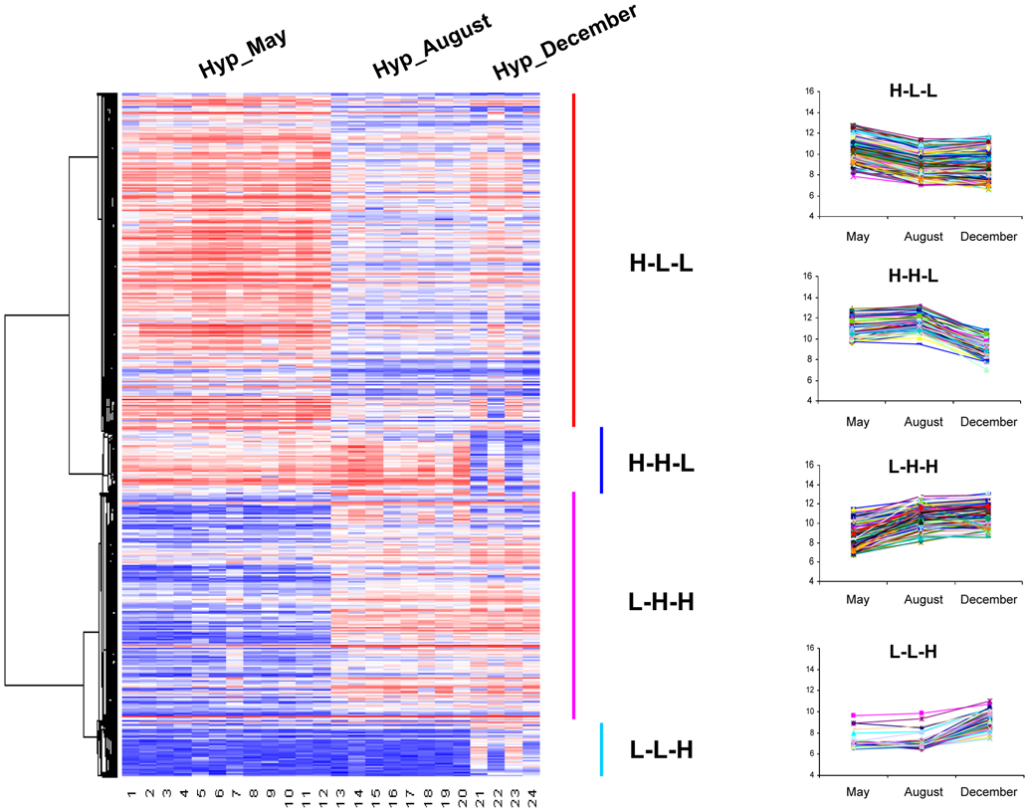
Hierarchical clustering of expression profiles of significantly differentially expressed genes between three reproductive seasonal time points (May, August, and December). Signal represents log ratio intensity of the gene expression. Red indicates relatively high expression and blue indicates relatively low expression.

We identified the differentially expressed genes between these three seasonal time points. The optimal discovery procedure (ODP) method [44] implemented in an EDGE program [45] was utilized for this purpose. A total of 873 unique differentially expressed genes were identified with high statistical significance (*q* value<0.0001) (Table S2). These differential genes constitute about 10% total genes (8448 genes) represented on the arrays and include 662 genes whose function is at least partially characterized and 211 unidentified EST sequences. All of the genes were further subjected to hierarchical cluster analysis (HCA) using Pearson correlation as a distance function. In the HCA, not only the relationships between different samples can be classified, but also the genes with similar expression patterns can be grouped by visual inspection of the hierarchical cluster results. As shown in Figure 2, the identified differentially expressed genes fall into four gene expression clusters comprising H-L-L, H-H-L, L-H-H and L-L-H (L, relatively low expression and H, relatively high expression) patterns along the seasonal cycle (May-August-December). Most of the identified genes belong to H-L-L and L-H-H expression clusters.

### Telencephalon exhibits similar transcriptomic patterns as hypothalamus

**Figure 3:**
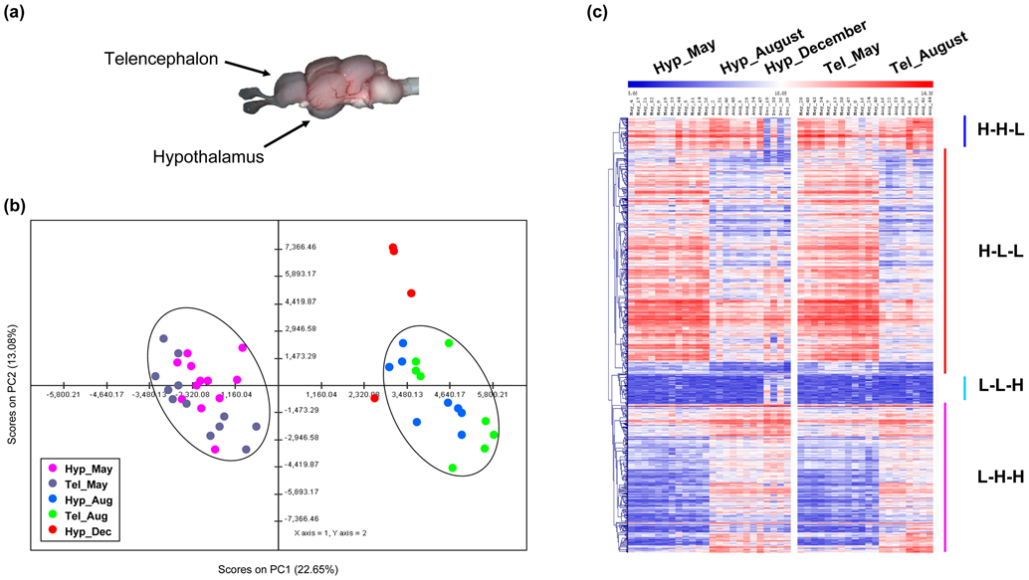
Similar transcriptomic patterns in both hypothalamus and telencephalon. (a) Location of hypothalamus (Hyp) and telencephalon (Tel) in goldfish brain. (b) Two-dimensional PCA plot for transcriptomes of both Hyp and Tel slides in three seasonal time points (May, August, and December). Eigenvalues of PCA are listed in Table S3. (c) Hierarchical clustering of the expression profiles of the significantly differentially expressed genes in Hyp and Tel along the seasonal cycle.

We next examined the global transcriptome similarity between Hyp and Tel during the seasonal cycle. Both Hyp and Tel are important brain regions involved in neuroendocrine control of growth and reproduction (Figure 3a). Five expression datasets (n = 20) for female Tel in May and August were available for this study. After the previously applied data normalizations, PCA was performed for both Hyp and Tel datasets. The output of PCA showed that transcriptomes of Hyp and Tel samples in both May and August are overlapped (Figure 3b). This indicates that these tissues have highly similar gene expression profiles in the same season (*e.g.* in May and August).

We further investigated whether the expression patterns of differentially expressed genes identified in Hyp are similar in the Tel. HCA was carried out on a combined data set including the expression values of all differential genes from Tel slides and those from the Hyp slides. We found that as in Hyp, those genes also exhibit clear differential expression patterns in Tel between May and August (Figure 3c). This was also observed by clustering global transcriptomes of both Hyp and Tel (not shown). Therefore, using multivariate analysis we illustrate that the transcriptomes from two neuroendocrine brain regions (Hyp and Tel) that are controlling reproduction are surprisingly similar within a given season and the differential gene patterns identified in Hyp was also evident in Tel.

### The identified gene expression patterns can be regulated by photoperiod

**Figure 4:**
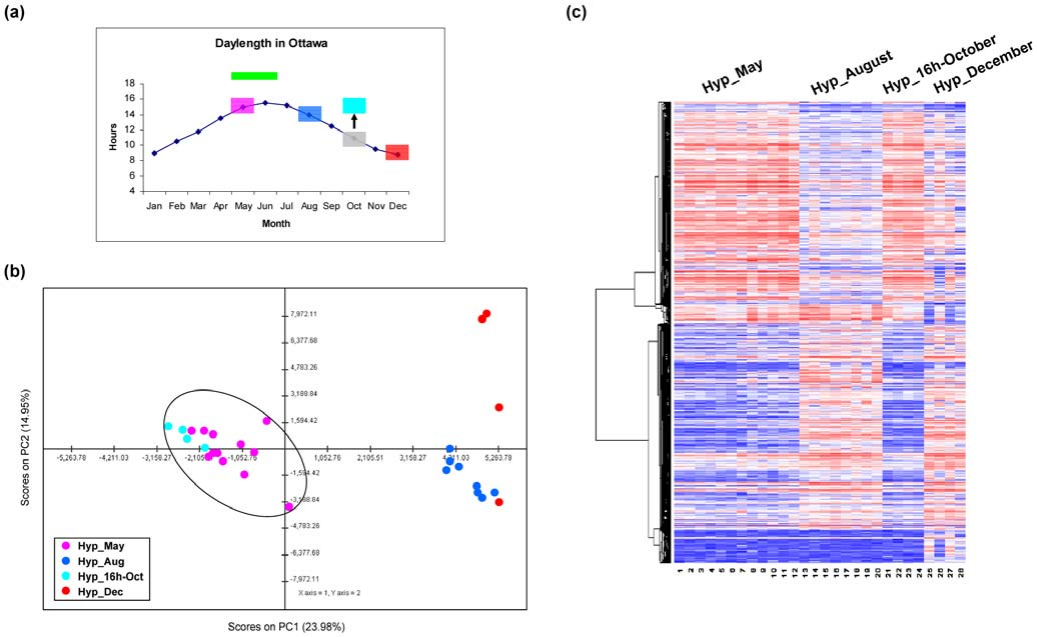
Regulation of brain gene expression patterns by photoperiod. (a) Seasonal profile of the daylength in Ottawa, Canada (45°27′N, 75°42′W). Pink, blue, grey and red boxes indicate daylength in May, August, October and December, respectively. The light blue box indicates a 16-hour daylength that we used for one fish group in October. Green bar indicates breeding season for goldfish typical in temperate North America. (b) Two-dimensional PCA plot for transcriptomes from Hyp slides in four seasonal time points (May, August, 16 h-October, and December). (c) Hierarchical clustering of expression profiles of significantly differentially expressed genes in Hyp slides along four seasonal time points.

Photoperiod is a major environmental factor that governs fish reproductive seasonality [3], [4], [5], [46]. Photoperiodic manipulations have been used to control the egg production and fertilization success of diverse species such as goldfish [47], [48], rainbow trout (*Salmo gairdneri*) [49], Japanese medaka (*Oryzias latipes*) [6] and European sea bass (*Dicentrarchus labrax L.*) [3]. Moreover, some photoperiod-regulated genes have also been described in the ovine pituitary gland [50] and the hamster hypothalamus [51]. Therefore, it can be hypothesized that the identified gene signature which is differentially expressed between seasons is regulated by photoperiod. One dataset provided a unique opportunity to clarify this question in which Hyp brain samples were taken from fish in October that had been acclimated to a long-daylength photoperiod (16 h), typical of the springtime breeding season in May (Figure 4a). The prediction was that if the identified gene expression signature is photo-responsive, the genes in 16 h-October fish should have similar expression patterns to fish in May acclimated for 3 weeks under our seasonally varying daylength protocol and sampled at 15.8 h light-length.

We first examined the relationship of the transcriptomes between the 16 h-October Hyp samples and other three normal Hyp samples using PCA. As shown in Figure 4b, the data points of 16 h-October slides are closely clustered with May slides, indicating similar global transcriptome status between the May and 16 h-October groups. We also compared the gene expression relationship of the identified differentially expressed genes in these four cases. As shown in Figure 4c, the expression of these 873 genes highly resembles that observed in May. Moreover, the gene expression patterns are reversed in 16 h-October compared to other seasons August and December. When we combined this dataset for a re-analysis to identify differential genes between seasons, we obtained a very similar gene list and gene patterns (data not shown). Therefore, we concluded that the identified differential expression patterns of these genes are likely regulated by photoperiod.

### Gene ontology analysis of differentially expressed genes

**Figure 5:**
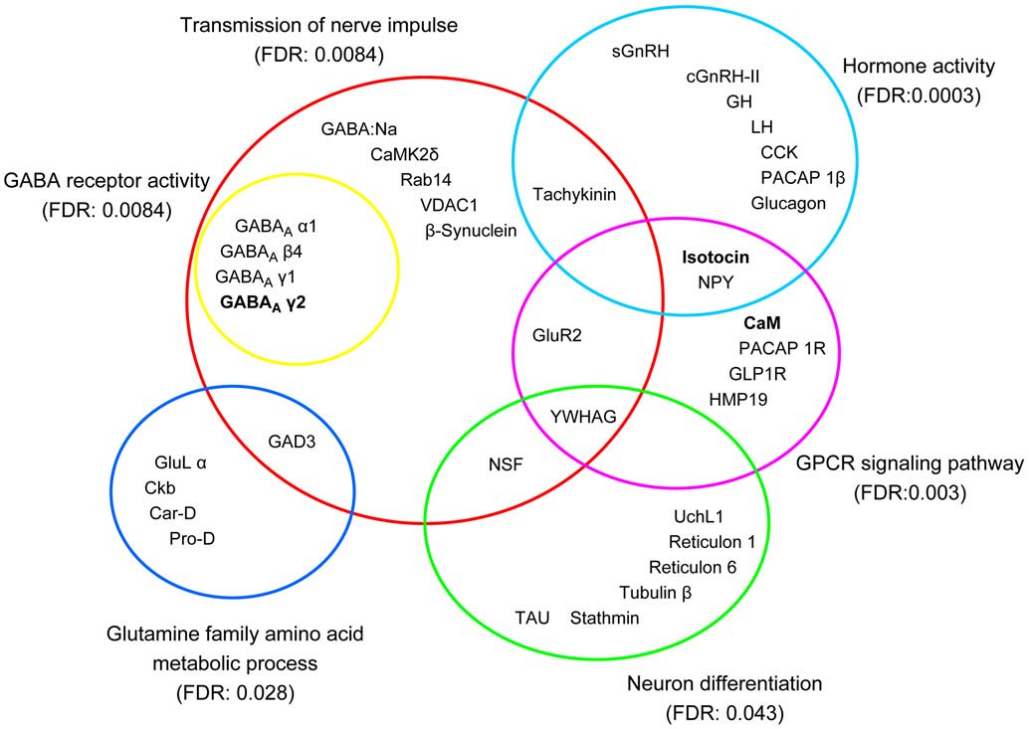
The neuroendocrine genes which are significantly enriched in the H-L-L expression pattern and their associated Gene Ontology categories. Gene abbreviations: CaM, calmodulin; CaMK2δ, calcium/calmodulin-dependent protein kinase (CaM kinase) II delta; Car-D, carnosine dipeptidase 1; CCK, cholecystokinin; cGnRH-II, chicken gonadotropin-releasing hormone II; Ckb, creatine kinase, brain; GABA_A_ α1, β4, γ1 and γ2, GABA_A_ receptor alpha1, beta4, gamma1 and gamma2; GABA:Na, GABA:Na symporter; GAD3, glutamate decarboxylase isoform 3; GH, growth hormone; GLP1R, Glucagon-like peptide-1 receptor; GluL α, glutamate-ammonia ligase alpha; GluR2, glutamate receptor, metabotropic 2; HMP19, neuron specific gene family member 2 (protein p19); LH, luteinizing hormone; NPY, Neuropeptide Y; NSF, n-ethylmaleimide-sensitive factor; PACAP 1β, pituitary adenlyate cyclase activating polypeptide 1 beta; PACAP 1R, PACAP type 1 receptor; Pro-D, Proline dehydrogenase; Rab14, member 14 of RAS oncogene family; sGnRH, salmon gonadotropin-releasing hormone; β-Synuclein, synuclein beta; TAU, microtubule-associated protein tau; UchL1, ubiquitin carboxyl-terminal esterase L1 (also called ubiquitin thiolesterase); VDAC1, voltage-dependent anion channel 1; YWHAG, 3-monooxygenase/tryptophan 5-monooxygenase activation protein, gamma polypeptide 2.

We have shown that a core set of genes were differentially expressed between seasons in both Hyp and Tel regions and they are photoperiod-responsive. We hypothesize that the affected genes should be related to neuroendocrine regulation of the fish reproductive cyclicity. We utilized GO term analysis to explore such potential functional significance among these genes. A series of GO categories implicated in neuroendocrine function are significantly over-represented for the genes of the H-L-L pattern (Figure S2). Figure 5 shows the primary GO categories with member genes, some of which can be found in multiple categories. Importantly, many genes have been functionally assigned to hormone activity, including two GnRH forms (salmon GnRH and chicken GnRH-II), growth hormone (GH), isotocin, LH, tachykinin, neuropeptide Y (NPY), pituitary adenlyate cyclase activating polypeptide 1 beta (PACAP 1β). Most of them, especially the GnRHs, are well-known and central to neuroendocrine control of reproduction. Another identified molecular function is GABA_A_ receptor activity. Four subunits, GABA_A_ alpha1, beta4, gamma1 and gamma2, were highly expressed in May. The GABA_A_ receptor is a pentameric protein that is a ligand-gated Cl^−^ ion channel whose activation by GABA or the specific GABA_A_ agonist muscimol results in rapid and sustained LH release in goldfish [52]. Many other genes identified are involved in several biological processes including: transmission of nerve impulse, glutamine family amino acid metabolic process, G-protein coupled receptor (GPCR) signaling pathway and neuron differentiation. Some genes are shared between these biological process and molecular function categories, among which transmission of nerve impulse is a central core linking other categories. Successful identification of a series of neuroendocrine function-related genes by GO enrichment analysis supports the biological implication of gene expression change of the H-L-L (May-August-December) gene pattern in reproductive seasonality. Moreover, since the timing of major expression change corresponds to the transition from prespawning/spawning to sexually regressed female goldfish, the high expression status of these genes around May, therefore, should be required for the fish spawning period.

### Seasonal goldfish brain sampling and real-time RT-PCR verifications

**Figure 6:**
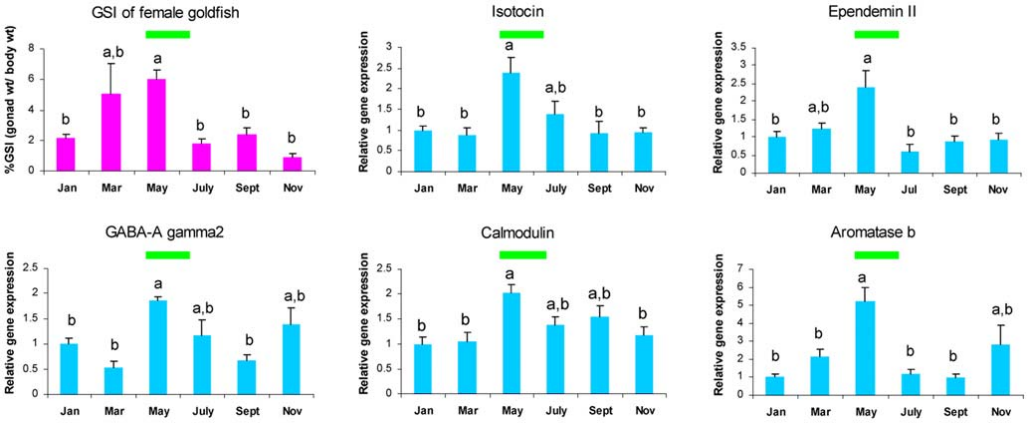
Seasonal profiles in GSI values and brain gene expression in female goldfish. (a) GSI values of female goldfish. (b)–(f) relative gene expression of neuroendocrine genes in female goldfish Hyp along a whole seasonal cycle. The green bar indicates the typical breeding period for goldfish in temperate North America.

Following bioinformatics analysis, we collected goldfish brain samples at six seasonal time points for experimental verification of gene expression patterns. Gonadosomatic index (GSI), an indication of the sexual maturity of the fish, shows variation along the reproductive season and reaches a peak around May (Figure 6a). The observed changes are comparable to previous studies in Japan [35] and Canada [24]. We used real-time RT-PCR to examine the seasonal expression profiles of five genes: isotocin, GABA_A_ gamma2, calmodulin, ependymin II and aromatase b. Among these genes, isotocin, GABA_A_ gamma2 and calmodulin are enriched in GO term analysis (Figure 5). Isotocin is a nine amino acid neuropeptide whose function is related to the control of reproductive and social behaviours in teleost fish [53], [54]. The effects on LH release by its mammalian homologue, oxytocin, was also reported in mammalian models [55], [56]. GABA_A_ gamma2 is one subunit of GABA_A_ receptor complex that is involved in binding of GABA_A_ receptor modulators such as benzodiazepines [57]; it is a representative of GABA system genes. Calmodulin is a calcium ion-binding protein and has diverse regulatory roles in L-type calcium channel, signal transduction, synthesis and release of neurotransmitters [58], and transcriptional action of estrogen receptors [59]. Ependymin II is a brain glycoprotein and is implicated in neuronal regeneration [60]. Aromatase b is the brain type aromatase, a P450 family enzyme expressed exclusively in teleost radial glial cells [61], and contributes to catalyzing the aromatization of testosterone (T) to 17beta-estradiol (E2). Although aromatase b is not identified by our meta-analysis, we are interested in it because both T and E2 have regulatory effect on the neuroendocrine system and the seasonal activity of aromatase b in fish brain is known to correlate significantly with seasonal reproductive cycles [23].

As shown in Figure 6b–f, seasonal expression profiles of these five genes in Hyp exhibit a clear increased expression in May. Significant differences (P<0.05; Holm-Sidak multiple test) can also be observed for the expression of most genes between May and other seasons. The range of overall fold changes for those genes (Figure 6) is similar to microarray analysis (Figure 2). Therefore, these results strongly support the patterns found in our meta-analysis of microarray data with an apparent expression peak in May representing the prespawning season of the goldfish.

## Discussion

An emerging concept about neuroendocrine regulation of biological rhythms is the recognized importance of the seasonal gene expression profiles in the neuroendocrine brain. Although several elegant studies have attempted to define gene expression change associated with hibernation in whole squirrel brain [62], migration in sparrow telencephalon [63] or photo-response in hamster hypothalamus [51], no system-level study has been conducted to profile the global gene expression changes along a whole reproductive cycle. The purpose of this study was to extract and evaluate such seasonal gene expression characteristics from the existing multiple microarray datasets using the goldfish model. A series of normalization procedures were utilized to remove systematic and experimental biases. The overall similarity among the slides from the same season as determined with PCA indicates that this strategy is effective. On the basis of this meta-type dataset, we identified four clear seasonal gene expression patterns in female goldfish brain. Interestingly, approximately 90% of genes represented on the array exhibit no detectable variation between the examined seasons. This suggests that the fish brain is a relatively stable system. The array does not cover the full transcriptome, but many biological pathways and processes are represented and they did not exhibit any detectable seasonal variation. In contrast, a set of genes were found differentially expressed between seasons and most of them fall into two distinct expression patterns (H-L-L and L-H-H). We observed that both female Hyp and Tel have a similar transcriptome status and same set of genes are differentially expressed between seasons. We further illustrated that artificially increasing daylength in October (16 h light) resulted in an expression profile in Hyp very similar to that of female goldfish in May (15.8 h light). This supports the hypothesis that photoperiod is a major environmental cue for vertebrate reproduction [4] because gene expression profiles in the neuroendocrine brain were photoperiodically-regulated to some degree.

Most differential genes showed expression changes between May and August in H-L-L and L-H-H patterns. May and August correspond to fish reproductive status of sexually mature prespawning and sexual regression respectively, and histological (ovary) and serum hormone changes have been extensively described between these two stages [23], [24], [28]. Thus, expression transition of this core gene set in the neuroendocrine brain may provide the molecular basis for phenotypic changes in the pituitary gland, circulating serum hormone levels and gonad status between seasons. Indeed, a series of GO categories related to neuroendocrine function and process are significantly overrepresented for the genes of H-L-L pattern (Figure 5). Many associated genes have been shown directly involved in regulating brain function and seasonal reproduction, as exemplified by the neuropeptides GnRH, isotocin, NPY and CCK. Moreover, other biological processes have also been identified including glutamate/GABA systems, GPCR signaling pathway and transmission of nerve impulse. Although no GO category is significantly enriched for the genes of L-H-H pattern, we speculate that they may include some inhibitory factors for neuroendocrine function such as the dopamine system related genes [12]. Other potential functions of these L-H-H genes may be involved in the marked seasonal growth phase of goldfish, which is different from reproductive phase [8]. We further verified the seasonal expression profiles for five genes which represent the GABA system (GABA_A_ gamma2), estrogen production (aromatase b), peptide hormones (isotocin), calcium binding proteins (ependymin II and calmodulin) and GPCR signaling pathway (calmodulin). Their seasonal expression profiles all exhibit similar predicted patterns with the apparent peak around May, thereby suggesting that a seasonal expression change is employed by their represented systems (*e.g.* E2 and GABA) to coincide with fish reproductive development.

Our study may serve as a basis for understanding reproductive seasonality of other fish species. Seasonal patterns in serum levels of reproductive hormones has been documented in many teleost species (*e.g.*, Florida gar (*Lepisosteus platyrhincus*) [36], masu salmon (*Onchorhynchus masou*) [64], and European sea bass [25]), including the goldfish [35]. It can be hypothesized that although different fish species have their own reproductive strategies and particular seasonal pattern, they may share one core set of genes with an increased expression prior to the spawning period. Indeed, similar increased expression pattern of several neuroendocrine genes has been observed in other fish species. For example, Okuzawa and colleagues [65] have shown that GnRHs exhibit a similar seasonal expression pattern in the brain of female red seabream (*Pagrus major*) with an expression peak around April, correlating with high steroid level and mature gonad state. Increased expression of GnRHs can also be found in the turbot (*Scophthalmus maximus*) [66], in parallel with an increase in oocyte diameter. Aromatase is another example; in the midshipman fish (*Porichthys notatus*), aromatase b in the magnocellular preoptic area of the brain showed higher expression in the pre-nesting period when compared to nesting and non-reproductive periods [67]. Moreover, in both red-spotted grouper (*Epinephelus akaara*) [68] and in the European sea bass [25], aromatase b showed enhanced activity during their own prespawning periods, which are corresponding to April and January, respectively.

Overall, using both bioinformatic and experimental strategies, we defined a core gene set whose expression exhibits a seasonal change along a fish reproductive cycle and illustrated that the gene expression change is primarily regulated by photoperiod. Moreover, most genes undergo an expression transition between May and August, which corresponds to the transition from prespawning to sexual regression. Thus the gene expression patterns may account for dynamic neuroendocrine regulation on fish seasonal reproductive development and the increased expression of these neuroendocrine related genes in May is crucial for ensuing spawning period. This is all the more critical in species with a short breeding period or where seasonally timed spawning occurs only once in the year.

## Materials and Methods

### Ethics statement

The care and treatment of animals used in this study were in accordance with the guidelines of the Animal Care Committee, University of Ottawa and the Canadian Council on Animal Care.

### Experiments and microarray datasets

All gene expression data were from version 1.0 Carp-Goldfish arrays which were printed in at University of Liverpool Microarray Facility, UK. The Gene Expression Omnibus (GEO) database accession number of the microarray platform is GPL3735. A total of 48 cDNA microarray slides were used for this study, and each slide was comprised of 6119 unique gene sequences and 2329 EST sequences printed in duplicate on each slide. Microarray datasets of gene expression in female goldfish hypothalamus and telencephalon have been collected to represent four seasonal time points. Detailed information about the experimental design, brain tissue, sampling seasonal time, temperature, daylength and other information are listed in Table S1. Some original results of corresponding experiments have been published [37], [39], and others will be published elsewhere. The hybridization, scanning procedures and validation of microarrays has been described previously by our group [37].

### Data normalizations

For each array, spots that had been manually flagged due to poor hybridization and spots in which the estimated fluorescence intensity was below or equal to the estimated background signal intensity in either channel were removed before further analysis. Lowess normalization [69] with span 0.4 was used to decrease the intensity dependent biases within slide, followed by Scale normalization [69]. Thereafter, control sample intensities were recovered and extracted to build a new series of expression data for all the slides. Following this, Quantile normalization [70] was used for across-slide normalization to minimize biases among different experiments.

### Identification of differentially expressed genes

An optimal discovery procedure (ODP) method implemented in the Extraction of Differential Gene Expression (EDGE) program [44] was used to assess the significance of differential expression of the genes between different seasonal time points. The false discovery rate for selected genes was monitored and controlled by calculating the *q* value. Genes with *q* value<0.0001 were considered significantly differentially expressed genes between comparison groups.

### Multivariate data analysis

Two multivariate methods including principal component analysis (PCA) and hierarchical clustering analysis (HCA) were utilized in this study. PCA was used to examine the transcriptome similarity relationship among slides that correspond to different seasonal time points. HCA was used to classify/cluster the gene expression patterns. Briefly, PCA combines two or more correlated factors (i.e. transcripts) into one new variable, a principal component (PC) [41], [42], [43]. In PCA the dimensionality of the dataset is reduced by replacing the original variables by a smaller number of newly formed variables that are linear combinations of the original variables and that explain the majority of the information (variability) from the experiment. Eigenvalues of PCA can be found in Table S3.

A hierarchical clustering tree [71] generates a binary dendrogram representing the association structure of pairs of arrays or genes, which was measured in terms of the Pearson correlation of the standardized intensity measurements. The algorithm identifies the gene pair with the smallest distance and groups them with a link, where distance is defined to be one-minus the correlation. The algorithm proceeds in a recursive manner to build the tree structure step by step. PCA and HCA were conducted by the MultiExperiment Viewer (MeV) [72] and EDGE [44] programs.

### Gene Ontology analysis

The resulting differentially expressed genes from each pattern were submitted to the Blast2GO web server [73]. Enrichment of multiple-level GO functional categories was determined using the GOSSIP/Fisher Exact test package [74], through a comparison of the categories found in our list of genes compared to the list of all genes found on the goldfish cDNA microarray. FDR controlled *p*-values (FDR<0.05) were used for the assessment of functional significance.

### Animal husbandry and tissue sampling

Adult female goldfish (*Carassius auratus*) were purchased from Aleong's International Inc. (Mississauga, ON, Canada), acclimated for 3 weeks to 18°C under a natural seasonally variable simulated photoperiod except for the animals sampled in October. In this case, animals were acclimated to 18°C and 16 h daylength that is typical of springtime. Fish were fed and maintained on standard flaked goldfish food. For the seasonal profile, 18 female fish were collected every two months starting in January, for a total of six time points (January, March, May, July, September, and November). Goldfish were anesthetized using 3-aminobenzoic acid methylester (MS222; Aquatic Eco-Systems, Apopka, FL) for all handling and dissection procedures. Care was taken to standardize all handling and sample protocols. Goldfish were sacrificed by spinal transection and 3 hypothalami were pooled for a total n = 6. Samples were rapidly dissected and immediately frozen on dry ice. At the time of sampling, body weights and gonadal weights were recorded and the gonadosomatic index (GSI; body weight/gonad weight ×100) was calculated.

### RNA extraction, quality assessment and cDNA synthesis

RNA was isolated using the RNeasy Plus Mini kit (Qiagen) as described in the manufacturer's protocol. Briefly, brain samples were homogenized using the stainless steel beads before using the RNeasy Plus Mini kit. Upon purification, concentration and quality of all samples was assessed using the 2100 Bioanalyzer (Agilent). RIN values of RNA samples ranged from 7 to 9.5, which is above the recommended minimum value of 5 for quantitative real-time RT PCR applications [75]. Total cDNA was prepared from 1 µg total RNA and 200 ng random hexamer primers (Invitrogen) using Superscript II RNase H- reverse transcriptase (SSII) as described by the manufacturer (Invitrogen). Each 20 µl reaction was diluted 10-fold in nuclease-free water and used as the template for the real-time RT-PCR assays. All brain cDNA samples from different seasonal time points were synthesized in parallel.

### Real-time RT-PCR verification

SYBR green real-time RT-PCR assay was used to validate relative expression of five genes (isotocin, GABA_A_ gamma2, calmodulin, ependymin II and aromatase b) at all six time points along the reproductive cycle. Primers (Table S4) were designed using Primer3 [76] and synthesized by Invitrogen. The Mx3000 Quantitative PCR System (Stratagene, La Jolla, CA) was used to amplify and detect the transcripts of interest. Each real-time RT-PCR reaction contained the following final concentrations: 25 ng first strand cDNA template, 1× QPCR buffer, 3.5 mM MgCl_2_, optimized concentrations (150 nM–300 nM) of gene-specific primers, 0.25× SYBRGreen (Invitrogen), 200 µM dNTPs, 1.25U HotStarTaq (Invitrogen), and 100 nM ROX reference dye, in a 25 µL reaction volume. The thermal cycling parameters were an initial 1 cycle Taq activation at 95°C for 15 min, followed by 40 cycles of 95°C for 15 s, optimized annealing temperature (58–60°C) for 5 s, 72°C for 30 s, and a detection step at 80°C for 8 s. Dilutions of cDNA (1∶10 to 1∶31,250) from all samples were used to construct a relative standard curve for each primer set. After the reaction was complete, a dissociation curve was produced starting from 55°C (+1°C/30 s) to 95°C. For each PCR reaction, negative controls were also introduced including a no template control (NTC) where RNase-free water was added to the reaction instead of the template (cDNA) and a no reverse transcriptase control (NRT) where RNase-free water was added to the cDNA synthesis reaction (previously described) instead of the SSII enzyme. The SYBR green assay for every target gene was optimized for primer concentration and annealing temperature to obtain a dRn value higher than 0.98, an amplification efficiency between 90%–110% and a single sequence-specific peak in the dissociation curve.

Data were analyzed using the MxPro software package. The relative standard curve method was used to calculate relative mRNA abundance between samples, which was then presented as means+SEM of gene expression from six biological replicates (assayed in duplicate) for each time point. A one-way analysis of variance (ANOVA) followed by a Holm-Sidak post-hoc test in SigmaStat3.5 (SPSS Inc.) was used to evaluate significant changes in gene expression between different time points. All data were first tested for normality and those data with non-normal distribution were subjected to square root transformations prior to statistical analyses.

## Supporting Information

Figure S1 Two-dimensional PCA plot for transcriptomes from Hyp slides in three seasonal time points (May, August, and December).
(0.08 MB PPT)

Figure S2 Gene ontolgy (GO) term enrichment analysis for the genes with H-L-L pattern.
(0.28 MB PPT)

Table S1 Detailed information about the experimental design, brain tissue, sampling seasonal time and other information.
(0.04 MB DOC)

Table S2 A full list of differentially expressed genes between seasons.
(0.65 MB XLS)

Table S3 Eigenvalues of PCA for Figure 3.
(2.07 MB TIF)

Table S4 Oligonucleotide primers used for real-time RT-PCR assays.
(0.03 MB DOC)
