# Supplementary figures and images for: Defining Global Neuroendocrine Gene Expression Patterns Associated with Reproductive Seasonality in Fish
Source: PLoS One. 2009 Jun 5;4(6):e5816. doi: 10.1371/journal.pone.0005816 (PMC2686097; doi:10.1371/journal.pone.0005816)

## Slide 1
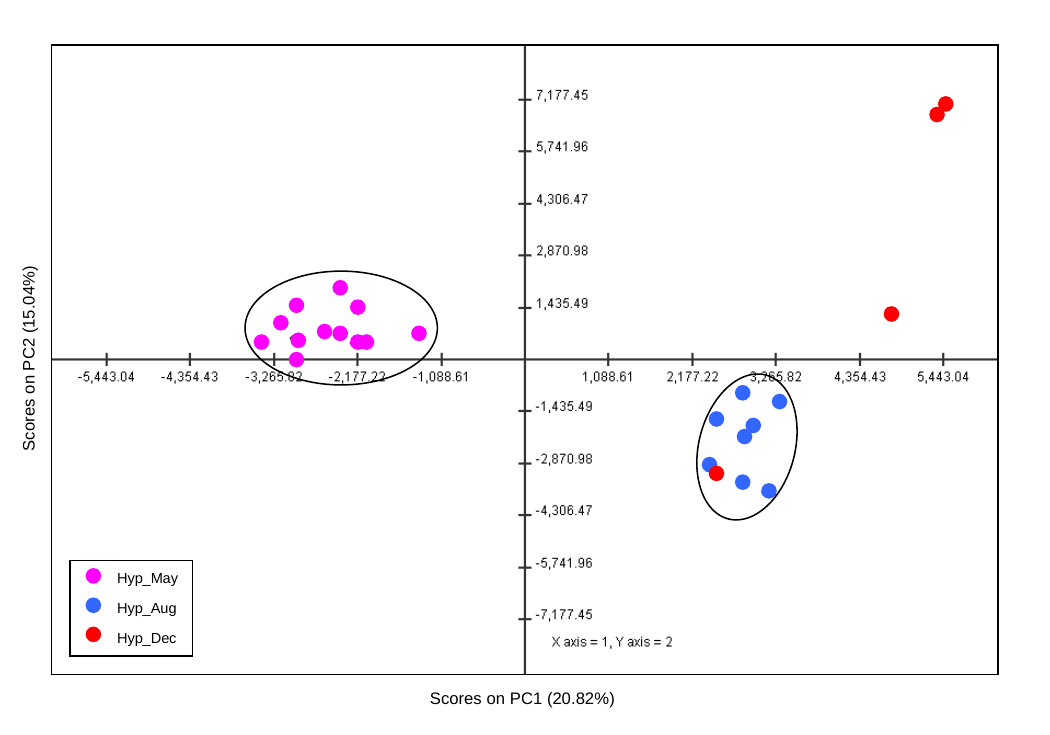

Scores on PC2 (15.04%)
Hyp_May
Hyp_Aug
Hyp_Dec
Scores on PC1 (20.82%)

Supplement: Figure S1 — Two-dimensional PCA plot for transcriptomes from Hyp slides in three seasonal time points (May, August, and December). (0.08 MB PPT) [file pone.0005816.s001.ppt]

## Slide 1
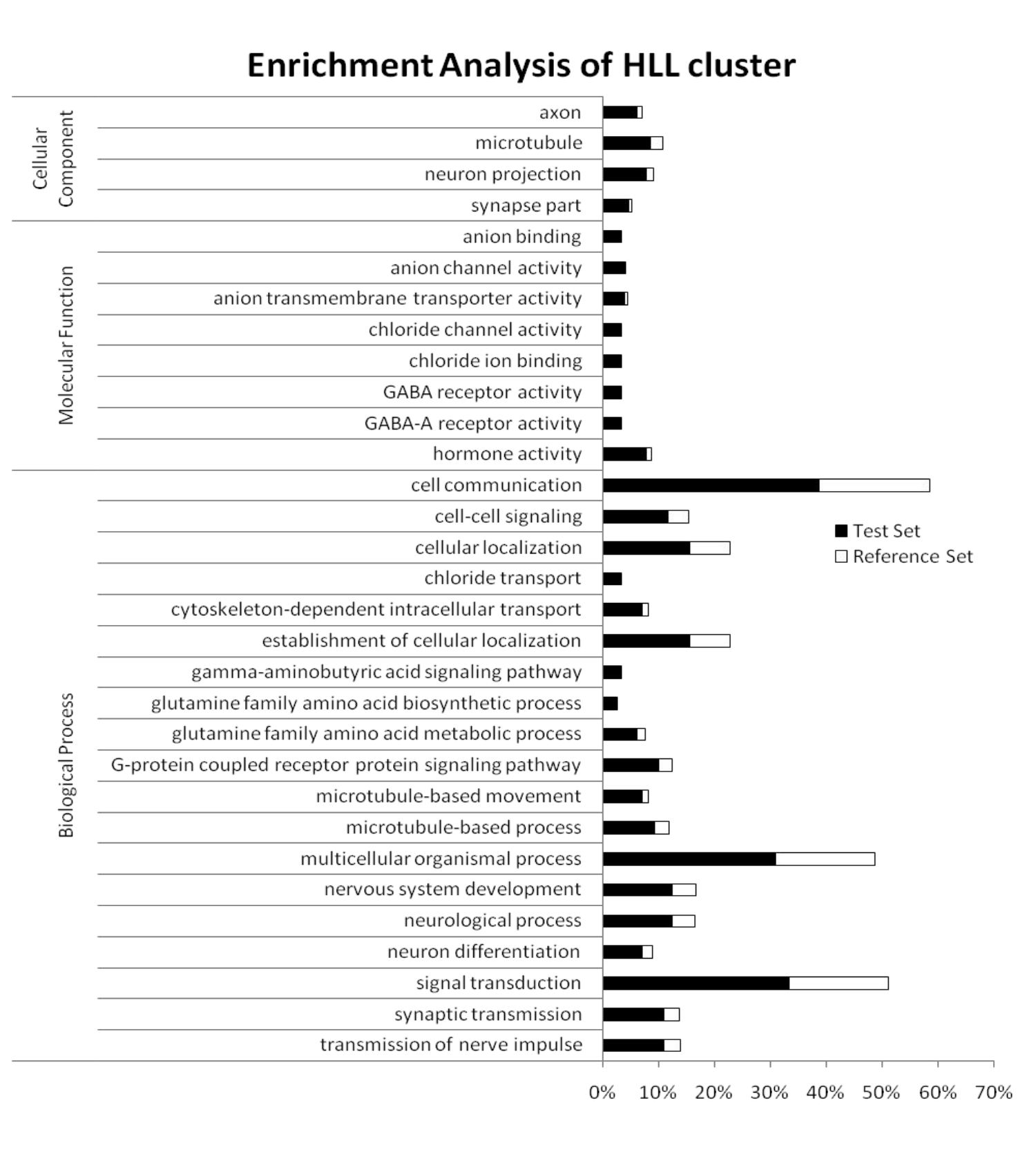

Supplement: Figure S2 — Gene ontolgy (GO) term enrichment analysis for the genes with H-L-L pattern. (0.28 MB PPT) [file pone.0005816.s002.ppt]

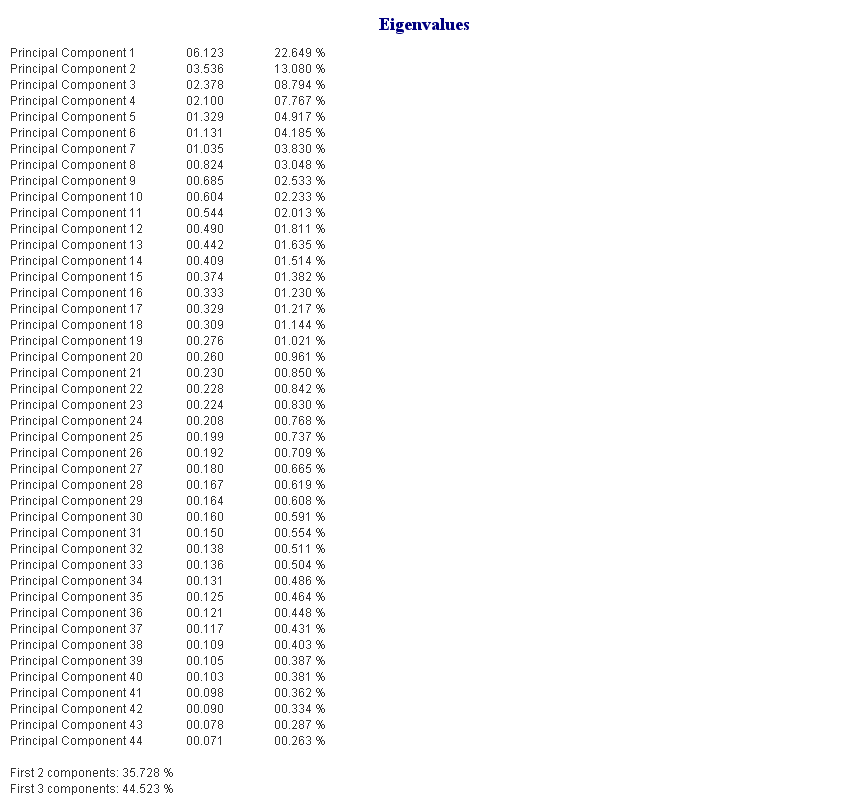

Supplement: Table S3 — Eigenvalues of PCA for Figure 3. (2.07 MB TIF) [file pone.0005816.s005.tif]
