# Supplementary material for: Defining Global Neuroendocrine Gene Expression Patterns Associated with Reproductive Seasonality in Fish
Source: PLoS One. 2009 Jun 5;4(6):e5816. doi: 10.1371/journal.pone.0005816 (PMC2686097; doi:10.1371/journal.pone.0005816)
Supplement: Table S1 — Detailed information about the experimental design, brain tissue, sampling seasonal time and other information. (0.04 MB DOC) [file pone.0005816.s003.doc]

**Additional file 1 - Detailed information about the experimental design, brain tissue, sampling seasonal time and other information**

| **Month** | **Sex** | **Tissue** | **Chemical** | **Sampling time** | **Gonad state** | **GEO series ID^c^** |
| --- | --- | --- | --- | --- | --- | --- |
| May | Female | Tel | MPTP^a^+αMPT^b^ | Mid-May 2004 | Prespawning | As |
| May | Female | Hyp | MPTP^a^+αMPT^b^ | Mid-May 2004 | Prespawning | As |
| May | Female | Hyp | D1R agonist, SKF 38393 | Early May 2006 | Prespawning | As |
| May | Female | Tel | D1R agonist, SKF 38393 | Early May 2006 | Prespawning | As |
| May | Female | Tel | D2R agonist, quinpirole | Mid-May 2006 | Prespawning | As |
| May | Female | Hyp | D2R agonist, quinpirole | Mid-May 2006 | Prespawning | As |
| August | Female | Tel | GABA_A_ agonist, muscimol | Late Aug. 2004 | Intact, sexually regressed | GSE5419 |
| August | Female | Hyp | GABA_A_ agonist, muscimol | Late Aug. 2004 | Intact, sexually regressed | GSE5768 |
| August | Female | Hyp | GABA_B_ agonist, baclofen | Early Sep. 2004 | Intact, sexually regressed | As |
| August | Female | Tel | GABA_B_ agonist, baclofen | Early Sep. 2004 | Intact, sexually regressed | As |
| October | Female | Hyp | MeHg | Oct. 2004 | Early redevelopment | As |
| December | Female | Hyp | Fluoxetine | Mid-Dec. 2004 | Early redevelopment | GSE5420 |

^a^1-methyl-4-phenyl-1,2,3,6-tetrahydropyridine; a selective dopaminergic neurotoxin

^b^α-methyl-*p*-tyrosine; a tyrosine hydroxylase inhibitor

^c^As, available soon
