# Supplementary material for: Defining Global Neuroendocrine Gene Expression Patterns Associated with Reproductive Seasonality in Fish
Source: PLoS One. 2009 Jun 5;4(6):e5816. doi: 10.1371/journal.pone.0005816 (PMC2686097; doi:10.1371/journal.pone.0005816)
Supplement: Table S4 — Oligonucleotide primers used for real-time RT-PCR assays. (0.03 MB DOC) [file pone.0005816.s006.doc]

**Additional file 4 - Oligonucleotide primers used for real-time RT-PCR assays**

| **Gene target** | **GeneBank ID** | **Sequence 5'-3'** | **Amplicon size** |
| --- | --- | --- | --- |
| Isotocin | AF322651 | F: GTATCTGCTGTGGTGAAGGT | 239 bp |
|  |  | R: ATCTTGGCTACTGGCAGCTT |  |
| Ependymin II | J04986 | F: TGATGCGGAACAATGAAAGTG | 258 bp |
|  |  | R: TCAGACTCGTGAGTGGCATA |  |
| GABA_A_ gamma2 | AY640227 | F: AGCTGGCACTCTGCATCAA | 173 bp |
|  |  | R: CTGCGTCTCAACAGCAACA |  |
| Calmodulin | AY656699 | F: CATTTCCATCAGCGTCCA | 108 bp |
|  |  | R: GCACCATCACGACCAAAGA |  |
| Aromatase b | AB009335 | F: TGCTGACATAAGGGCAATGA | 153 bp |
|  |  | R: GGAAGTAAAATGGGTTGTGGA |  |
